# Supplementary material for: Predicting the aggravation of coronavirus disease-19 pneumonia using chest computed tomography scans
Source: PLoS One. 2022 Nov 3;17(11):e0276738. doi: 10.1371/journal.pone.0276738 (PMC9632883; doi:10.1371/journal.pone.0276738)
Supplement: Attachment — Submitted filename: Response to reviewers.docx [file pone.0276738.s003.docx]

Response to Reviewers' comments:

Please use the space provided to explain your answers to the questions above. You may also include additional comments for the author, including concerns about dual publication, research ethics, or publication ethics. (Please upload your review as an attachment if it exceeds 20,000 characters)

Reviewer #1: Summary

Goal of this paper is trying to identify predictors for COVID-19 aggravation in a retrospective clinical analysis. Chest CT scan done by 15 days after disease onset is the key target for this study. 189 candidates were enrolled and divided into SPG(n=76) and NSPG(n=113) groups. It is concluded that the degree of lung damage detected by chest CT scanning in the early stage (3 days after onset) could serve as a predictor for disease aggravation.

⇒We thank the reviewer for the advice.

Major Issues

(1)

Line 108 : “The severe pneumonia group (SPG) included patients with intubation or respiratory management and those who died.”

-----

1. The definition of “respiratory management” is not precise enough. Does it include oxygen therapy, conventional ventilator therapy, NIPPV, HFNC, prone therapy and/or ECMO?

⇒The definition of "respiratory management" is ventilator management.

Therefore, the definition of "severe pneumonia" in this study is ventilator management or death.

It is described in lines 143-144.

The severe pneumonia group (SPG) included patients with intubation and conventional ventilator therapy or death.

2. In addition to severe pneumonia with acute respiratory failure, COVID-19 victims may also die of sepsis, cardiac dysfunction, thrombosis or other comorbidities. These cases will be recruited into the SPG group despite their lungs are less damaged, and that will distort the results of chest CT analysis.

⇒ "Myocardial infarction" and "pulmonary embolism" are excluded in this study.

It is described in lines 145-146.

Patients who were intubated for conditions other than pneumonia were excluded from the evaluations.

It is described in lines 186-188.

Intubation was performed for myocardial infarction in 3 patients, pulmonary embolism in 4 patients, and gastrointestinal perforation in 1 patient, and thus these patients were excluded from this study.

(2)

Line 129 : “Analysis of chest CT scans”

-----In this study the methodology of chest CT analysis adopted a scoring system from Muller NL (ref 10) which was specifically for pulmonary sarcoid analysis in 1989. The details of estimation method was not given in this paper. Nevertheless, new expert consensus and chest CT scoring system specifically for COVID -19 infections were available in 2020, which will be more specific and updated for your analysis.

Ref:

\# Marco Francone, Franco Iafrate, Giorgio Maria Masci, et al. Chest CT score in COVID-19 patients: correlation with disease severity and short-term prognosis. European Radiology 2020;30(12):6808-6817.

\# Feng Pan, Tianhe Ye, Peng Sun, et al. Time Course of Lung Changes at Chest CT during Recovery from Coronavirus Disease 2019 (COVID-19). Radiology 2020; 295:715–721.

\# Simpson S, Kay FU, Abbara S, Bhalla S, Chung JH, Chung M, Henry TS, Kanne JP, Kligerman S, Ko JP, Litt H. Radiological Society of North America expert consensus statement on reporting chest CT findings related to COVID-19. Endorsed by the Society of Thoracic Radiology, the American College of Radiology, and RSNA - secondary publication. J Thorac Imaging 2020;35:219-27.

\# Geoffrey D. Rubin, Christopher J. Ryerson, Linda B. Haramati, et al. The Role of Chest Imaging in Patient Management during the COVID-19 Pandemic: A Multinational Consensus Statement from the Fleischner Society. Radiology 2020; 296:172–180.

⇒The CT severity scoring of 2020 is had scoring of for each lobe. Therefore the right middle lobe of approximately 10% of total lung is actually estimated as 20% by CT severity scoring. As CT severity scoring was not exact for an area, we chose a standard method.

However, in consideration of the need of the on-site physician who used CT severity scoring, we wrote score jointly.

It is described in lines 171-175.

CT severity scoring [11] was calculated per each of the 5 lobes considering the extent of involvement, as follows:

0, no involvement; 1, < 5% involvement; 2, 5-25% involvement; 3, 26-50% involvement; 4, 51-75% involvement; and 5, > 75% involvement. The CT severity score was the sum of each individual lobar score (0 to 25).

It is described in lines 226-227.

The ROC curve for CT severity score demonstrated a sensitivity of 83.3%, specificity of 62.8%, and cut-off value of 9.

It is described in lines 287-289.

In the ROC curve for CT severity score, the sensitivity, specificity, and cut-off values were 70.6%, 75.0%, and 6 in the T2 period; 76.4%, 67.1%, and 9 in the T3 period; and 90.9%, 83.3%, and 10 in the T4 period.

(3)

Line 202-224 : “Analysis according to site”, “…..in terms of the area with damage combining GGO and consolidation in the upper, middle, and lower lobes (Fig 3A).”

-----According to your methodology the so called “the upper, middle, and lower lobes” in this study actually are not the same the terminology of upper, middle, and lower lobes in lung anatomy. Readers will get confused if your “lobe” is actually not a lobe, but a region in the lung.

⇒The expression "lobe" and “site” has been changed to "regions".

Minor Issues

Line 225-232 : “Clinical course analysis” “Figure 4A”

----- Simple linear regressions were conducted separately for both SPG and NSPG for different CT lesions. Please specif the y Y and X variables in the liner regression equation.

⇒In Figure 4a, we added Y = 2.822 × X + 20.18 as SPG and Y = 1.353 × X + 11.93 as NSPG.

In Figure 5b, we added Y = 2.750 × X + 23.27 as SPG and Y = 1.139 × X + 13.51 as NSPG

Other Comment

Line 73: “several pneumonia phases”

----- “severe pneumonia” or “several pneumonia” ?

⇒The expression " several pneumonia" has been changed to " severe pneumonia” in line 107

Line 93 :

“As our institution receives patients with moderate to severe pneumonia caused by COVID-19, factors that can predict aggravation from moderate to severe illness in patients who demonstrate the same were examined.”

----- “who demonstrate the same” ???

⇒In 126, we added

“Therefore, since our institution accepts patients with moderate to severe pneumonia by COVID-19, we investigated what predictors of severe disease were present in patients who consequently transitioned from moderate to severe disease.”

Line 98 : “was qualitatively examined.”

----- qualitatively or quantitatively or semi-quantitatively examined in this study?

In line132, we changed as “was examined qualitatively and quantitatively.”migi

Line 123 : “KL6 levels”

----- KL6 is not a popular abbreviation. Please provide full name in front of abbreviation.

⇒In line156, we changed as “sialylated carbohydrate antigen KL-6”

Line 124: “and the use of non-inhaled steroids, including favipiravir and remdesivir”

----- Favipiravir and remdesivir are actually anti-viral agents, not non-inhaled steroids.

Inlind157, we added “The history of use of inhaled steroids including Ciclesonide, non-inhaled steroids, favipiravir, and remdesivir was evaluated as factors affecting the severe pneumonia group.”

Line 290 310: “It has been highlighted that COVID-19 is an asymptomatic infection”

-----

⇒In Line 349, we changed to “It has been highlighted that COVID-19　is often asymptomatic and is difficult to control.”

Reviewer #2: This is a retrospective study. Although 277 COVID-19 patients were accessed initially, 65 patients were excluded because they had no computerized tomography, 8 patients were excluded because they had no pneumonia presented at chest CT scans. Fifteen patients were excluded due to clinical courses were unclear, so only 189 patients were included for course analysis. Given the study designs are not prospective cohort setting, there are some concerns for the interpretation of the results:

⇒We thank the reviewer for the advice.

Major concern 1: “Age” is an aggravation factors for most of the critical illness, according to existing reports, there was an insignificant difference between the SPG and NSPG in terms of age. The authors should address further analysis of the interaction between age factor and the presence or absence of underlying illnesses.

Figure 5 separately analyzes the individuals who were more than 65 years old, with significant differences. The readers must also request to see this comparison in younger group.

⇒In line 220, we added “Patients 65 years or older were more frequent of associated disease than younger patients (90.3% vs 65.2%).”

In line 299, we added “As data under 65 years old, there was not a significant difference observed in the chest CT scans in the SPG and NSPG,”

In line 304, we added “The data under 65 years old did not significantly have linear regression in the SPG and NSPG.,”

Major concern 2:

There should be other chest computerized tomography image findings, such as interstitial infiltration, septal thickening, cavitating lung lesions, or pneumothorax, etc. The author should try their bet to evaluate possible prognosticator among them.

⇒ These findings were rare and did not make a significant difference. Therefore the extent of consolidation and GGO was measured, and the mean was calculated.

Major concern 3:

Compared to COVID-19 patients with NSPG, those with SPG overall had higher GGO and consolidation scores; however, their values were overlapping seriously. This makes the results only provide us some insight in realizing pneumonia degree and extension correlate with clinical severity. It will be more practical to develop a chest computerized tomography scoring system to predict clinal outcomes. Please refer to the published paper “Chest CT Severity Score: An Imaging Tool for Assessing Severe COVID-19” at Radiology: Cardiothoracic Imaging Volume 2: Number 2, 2020.

⇒The CT severity scoring of 2020 is had scoring of for each lobe. Therefore the right middle lobe of approximately 10% of total lung is actually estimated as 20% by CT severity scoring. As CT severity scoring was not exact for an area, we chose a standard method.

However, in consideration of the need of the on-site physician who used CT severity scoring, we wrote score jointly.

It is described in lines 171-175.

CT severity scoring [11] was calculated per each of the 5 lobes considering the extent of involvement, as follows:

0, no involvement; 1, < 5% involvement; 2, 5-25% involvement; 3, 26-50% involvement; 4, 51-75% involvement; and 5, > 75% involvement. The CT severity score was the sum of each individual lobar score (0 to 25).

It is described in lines 226-227.

The ROC curve for CT severity score demonstrated a sensitivity of 83.3%, specificity of 62.8%, and cut-off value of 9.

It is described in lines 287-289.

In the ROC curve for CT severity score, the sensitivity, specificity, and cut-off values were 70.6%, 75.0%, and 6 in the T2 period; 76.4%, 67.1%, and 9 in the T3 period; and 90.9%, 83.3%, and 10 in the T4 period.

Minor concern 1:

Since the 65 patients without CT scans and the 8 patients without pneumonia presented at chest CT scans were excluded, the title should be verified as “The aggravation of coronavirus disease-19 pneumonia in chest computed tomography scans”.

⇒We changed the title to “The aggravation of coronavirus disease-19 pneumonia in chest computed tomography scans”

Minor concern 2:

Figure 2 to Figure 5 should annotate the longitudinal axis meaning to indicate what kind of percentage. Figure 2A lacks horizontal bar to indicate mean values.

⇒ We added “Lung damage volume (%)” in Figure 2 to Figure 5
