# Supplementary material for: Predicting the aggravation of coronavirus disease-19 pneumonia using chest computed tomography scans
Source: PLoS One. 2022 Nov 3;17(11):e0276738. doi: 10.1371/journal.pone.0276738 (PMC9632883; doi:10.1371/journal.pone.0276738)
Supplement: Attachment — Submitted filename: Response to Reviewers.docx [file pone.0276738.s004.docx]

31 May 2022

Dear Wen-Wei Sung, M.D., Ph.D.

Re. PONE-D-21-37765R1

Predicting the aggravation of coronavirus disease-19 pneumonia using chest computed tomography scans

On behalf of the authors, please find attached the above revised manuscript, with reviewer comments addressed, for consideration for publication in PLOS ONE

We would like to thank the reviewers for their feedback and have provided a point-by-point response to each of their comments in the table at the end of this letter.

We hope that their concerns have been addressed to your satisfaction and that the manuscript is now suitable for publication. We can confirm that this manuscript remains unpublished elsewhere and is not under consideration for publication elsewhere.

Thank you for your consideration of this manuscript and we look forward to receiving your decision soon.

Yours sincerely,
Yukitaka Yamazaki, on behalf of the authors

Corresponding author

Seido Ooka, MD., PhD

St. Marianna University School of Medicine,
2-16-1 Sugao Miyamaeku Kawasaki-city, Japan 216-8511

E-mail: ooka@marianna-u.ac.jp

Thank you very much for your reviewing. We totally agree to your suggestions and revised the text. Revised portions were colored red. We believe that this revision made the text better than before. Please, consider my article for publication in your journal.

Sincerely,

Yukitaka Yamazaki, Seido Ooka

Reviewers' comments:

Reviewer #1:

Line 205 Table 1
The superscript characters of “a” and “b” represent “number” and “standard deviation” respectively in Table 1. However, there are already abbreviations of “n” and “SD” to represent “number” and “standard deviation” in the same table. The superscript characters of “a” and “b” are superfluous.

⇒We thank the reviewer for the advice.

I deleted the superscript characters of "a" and "b"

Line 215 Table 2
The superscript characters of “a”, “b” and “c” represent “not significant” and “number” and “standard deviation” respectively in Table 2. However, there are already abbreviations of “n.s.”, “n” and “SD” in the table. The superscript characters of “a”, “b” and “c” are superfluous.
⇒We thank the reviewer for the advice.

I deleted the superscript characters of "a" and "b"

Line 251
“middlefield” →“middle field”

⇒We thank the reviewer for the advice.

I corrected it according to your advice.

Line 268
“The time point when a significant difference would appear in findings in CT scans conducted after the day of onset was examined.” →“The time point (X) when a significant difference would appear in findings in CT scans conducted after the day of onset was examined. ”
⇒We thank the reviewer for the advice.

I corrected it according to your advice.

Line 269
“ In the area with damage combining GGO and consolidation,” →“In the area with damage(Y) combining GGO and consolidation,”

⇒We thank the reviewer for the advice.

I corrected it according to your advice.
